# Neuroimaging in acute infection-triggered encephalopathy syndromes

**DOI:** 10.3389/fnins.2023.1235364

**Published:** 2023-08-10

**Authors:** Jun-ichi Takanashi, Hiroyuki Uetani

**Affiliations:** ^1^Department of Pediatrics, Tokyo Women’s Medical University Yachiyo Medical Center, Yachiyo, Japan; ^2^Diagnostic Radiology, Faculty of Life Sciences, Kumamoto University, Kumamoto, Japan

**Keywords:** magnetic resonance imaging, MR spectroscopy, diffusion-weighted imaging, arterial spin labeling, acute encephalopathy with biphasic seizures and late reduced diffusion, acute necrotizing encephalopathy, clinically mild encephalitis/encephalopathy with a reversible splenial lesion

## Abstract

Acute encephalopathy associated with infectious diseases occurs frequently in Japanese children (400–700 children/year) and is the most common in infants aged 0–3 years. Acute encephalopathy is classified into several clinicoradiological syndromes; acute encephalopathy with biphasic seizures and late reduced diffusion (AESD) is the most common subtype, followed by clinically mild encephalitis/encephalopathy with a reversible splenial lesion (MERS) and acute necrotizing encephalopathy (ANE). Neuroimaging, especially magnetic resonance imaging (MRI), is useful for the diagnosis, assessment of treatment efficacy, and evaluation of the pathophysiology of encephalopathy syndromes. MRI findings essential for diagnosis include delayed subcortical reduced diffusion (bright tree appearance) for AESD, reversible splenial lesions with homogeneously reduced diffusion for MERS, and symmetric hemorrhagic thalamic lesions for ANE. We reviewed several MRI techniques that have been applied in recent years, including diffusion-weighted imaging for the characterization of cerebral edema, arterial spin labeling for evaluating cerebral perfusion, and magnetic resonance spectroscopy for evaluating metabolic abnormality.

## 1. Introduction

Acute encephalitis is caused by the direct invasion of some viruses, such as the herpes simplex virus or Japanese encephalitis virus. It is usually accompanied by increased inflammatory cells in the cerebrospinal fluid. Acute encephalopathy is the generic term for acute central nervous system dysfunction, characterized by acute onset of severe and long-lasting (over 24 h) disturbances of consciousness, and is caused by various agents, such as infection, hypoxia, metabolic disease, and hepatic or renal dysfunction. The pathological substrate of acute encephalopathy is diffuse or widespread, non-inflammatory brain edema. Unlike encephalitis, inflammatory cells are not usually found in the brain or cerebrospinal fluid in encephalopathies. In Japan, acute encephalopathy affects 400–700 children per year and is usually preceded by infection, most often by influenza virus (16%), human herpes virus (HHV) 6 and 7 (16%), and rotavirus (4%) (Mizuguchi et al., 2007; Kasai et al., 2020).

Over the past 30 years, pediatric neurologists in Japan have established and characterized new encephalopathy syndromes based on characteristic radiological findings and clinical features. These include acute necrotizing encephalopathy (ANE), acute encephalopathy with biphasic seizures and late reduced diffusion (AESD), and clinically mild encephalitis/encephalopathy with reversible splenial lesions (MERS). AESD was the most common (34%), followed by MERS (18%) and ANE (3%) (Kasai et al., 2020). Magnetic resonance imaging (MRI) is a sensitive technique for the diagnosis of encephalopathy, and diffusion-weighted imaging (DWI) is particularly useful for detecting early changes. Arterial spin labeling (ASL) and magnetic resonance spectroscopy (MRS) can non-invasively reveal cerebral perfusion and metabolism. In this article, we review imaging modalities useful in the diagnosis of acute encephalopathy and elucidation of the pathological mechanism, including AESD, MERS, and ANE.

## 2. Clinically applicable MRI technique for encephalopathy

### 2.1. DWI

DWI signals reflect differences in the movement of protons in water molecules and are useful for evaluating the molecular function and microarchitecture of the brain (Baliyan et al., 2016). DWI can be quantified using apparent diffusion coefficient (ADC) maps. The DWI signal increases due to a lack of proton movement in regions where Brownian movement is restricted (decreased ADC) by cellular or myelin membranes, that is, cytotoxic edema or intramyelinic edema. Diffusion tensor imaging is a sophisticated version of DWI, with six or more detections of proton movement. Within the brain, water molecules tend to exhibit preferential movement in specific directions following the underlying cellular structures, such as axons and myelination. This property is called anisotropy and is mapped using fractional anisotropy (FA) mapping. Areas with high FA values (close to 1.0) show strong directionality of water molecular movement along the white matter tracts. By analyzing the movement of water molecules throughout the brain, a map of these fiber tracts can be constructed using diffusion tensor imaging tractography.

#### 2.1.1. AESD

**Figure 1:**
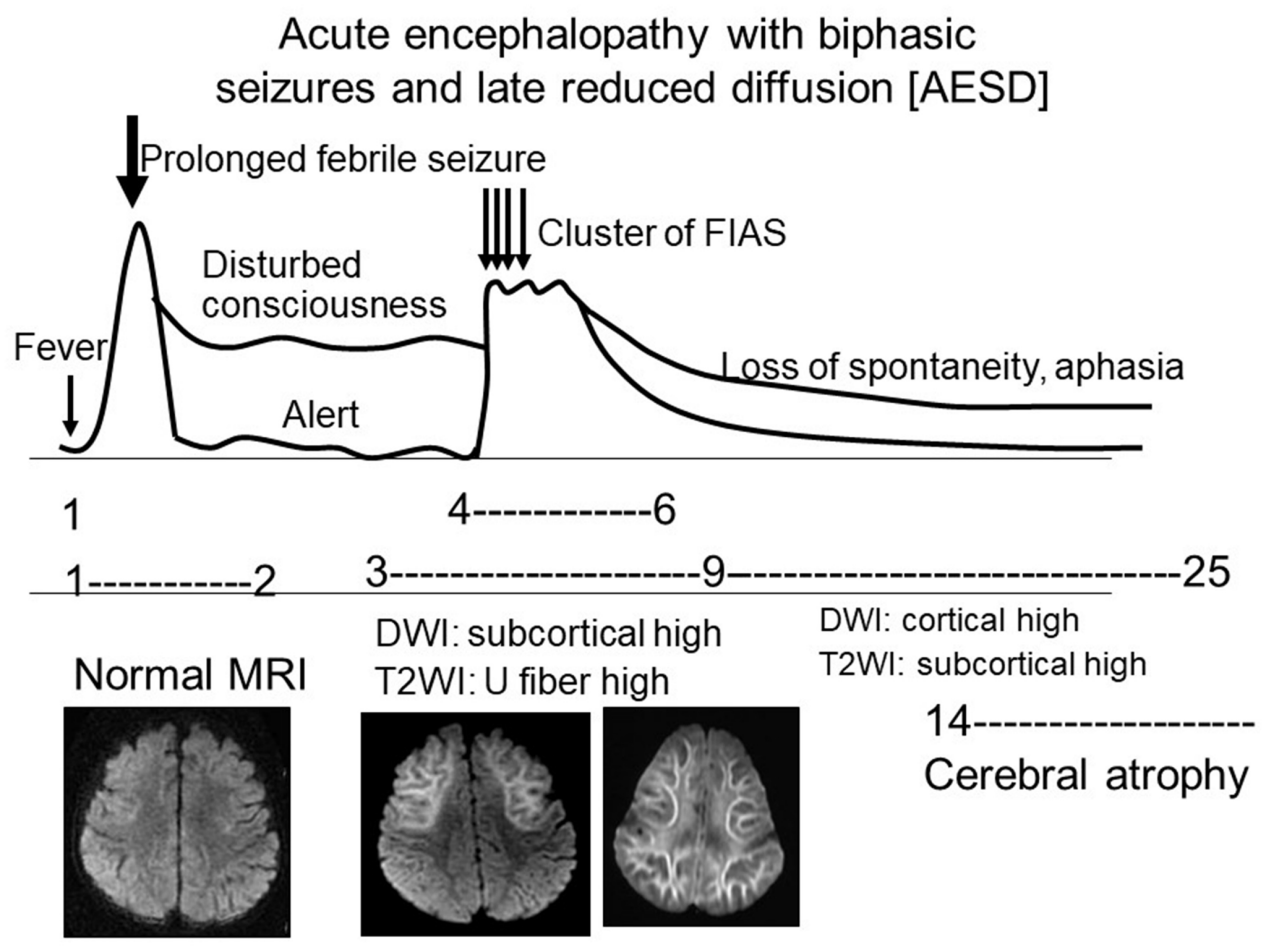
Clinical and radiological schema of AESD. This figure shows the temporal relationship between the seizures, level of consciousness, and MRI findings during the disease (from Takanashi, 2009a with permission). AESD, acute encephalopathy with biphasic seizures and late reduced diffusion; FIAS, focal impaired awareness seizures.

AESD is the most frequent encephalopathy syndrome in Japan (130–230/year), and most cases occur at around 1 year of age (Kasai et al., 2020). Only a few cases have been reported in countries other than Japan, which suggests the involvement of genetic factors. HHV 6 and 7 (32%) and influenza virus (7%) are the most common pathogens associated with AESD. AESD is more common in children with preexisting neurological abnormalities, and is clinically characterized by a biphasic course, that is, a prolonged febrile seizure (early seizure) on days 1–2, followed by late seizures, most often in clusters of focal impaired awareness seizures, associated with the deterioration of consciousness levels on days 4–6 (Takanashi et al., 2006a; Takanashi, 2009a; Mizuguchi et al., 2021) (Figure 1 and Table 1). The overall outcomes of patients with AESD have been reported to be almost normal (33%), with mild to moderate disability (43%), severe disability (19%), and death (2%). Neurological sequelae include intellectual and motor disabilities, and epilepsy, which is sometimes intractable.

**Table 1:** Magnetic resonance imaging (MRI) findings characteristic of encephalopathy syndromes (From Takanashi, 2018 with permission).

Acute encephalopathy with biphasic seizures and late reduced diffusion (AESD).
No abnormal lesion within 2 days. Diffusion-weighted imaging (DWI) revealed subcortical white matter lesions between days 3 and 9 (bright tree appearances). The lesions are predominantly frontal or frontoparietal, with sparing of the peri-rolandic region (central sparing). After 9 days, the bright tree appearance on DWI disappeared, and T2-weighted imaging (T2WI) or fluid-attenuated inversion recovery (FLAIR) imaging showed high-intensity lesions in the affected white matter, followed by cerebral atrophy. Magnetic resonance spectroscopy (MRS) showed an acute increase in glutamate (days 1–4), which changed to a subacute increase in glutamine (days 4–12). Decreased *N*-acetylaspartate (NAA) levels predict poor neurological outcomes.
Clinically mild encephalitis/encephalopathy with a reversible splenial lesion (MERS).
DWI shows a reversible lesion in the corpus callosum, at least in the splenium, with homogenously reduced diffusion and no contrast enhancement (type 1 MERS). A splenial lesion is sometimes associated with those in the symmetrical white matter, both of which are also reversible (type 2 MERS).
Acute necrotizing encephalopathy (ANE).
MRI shows diffuse cerebral edema and symmetric and multifocal lesions in the thalamus and other central nervous system regions, including the posterior limb of the internal capsule, posterior putamen, cerebral and cerebellar deep white matter, and the upper brainstem tegmentum. The thalamic lesions often show hemorrhagic degeneration and cystic changes after 3 days, showing a high signal on T1WI and a low signal on T2WI or T2 star-weighted imaging.

**Figure 2:**
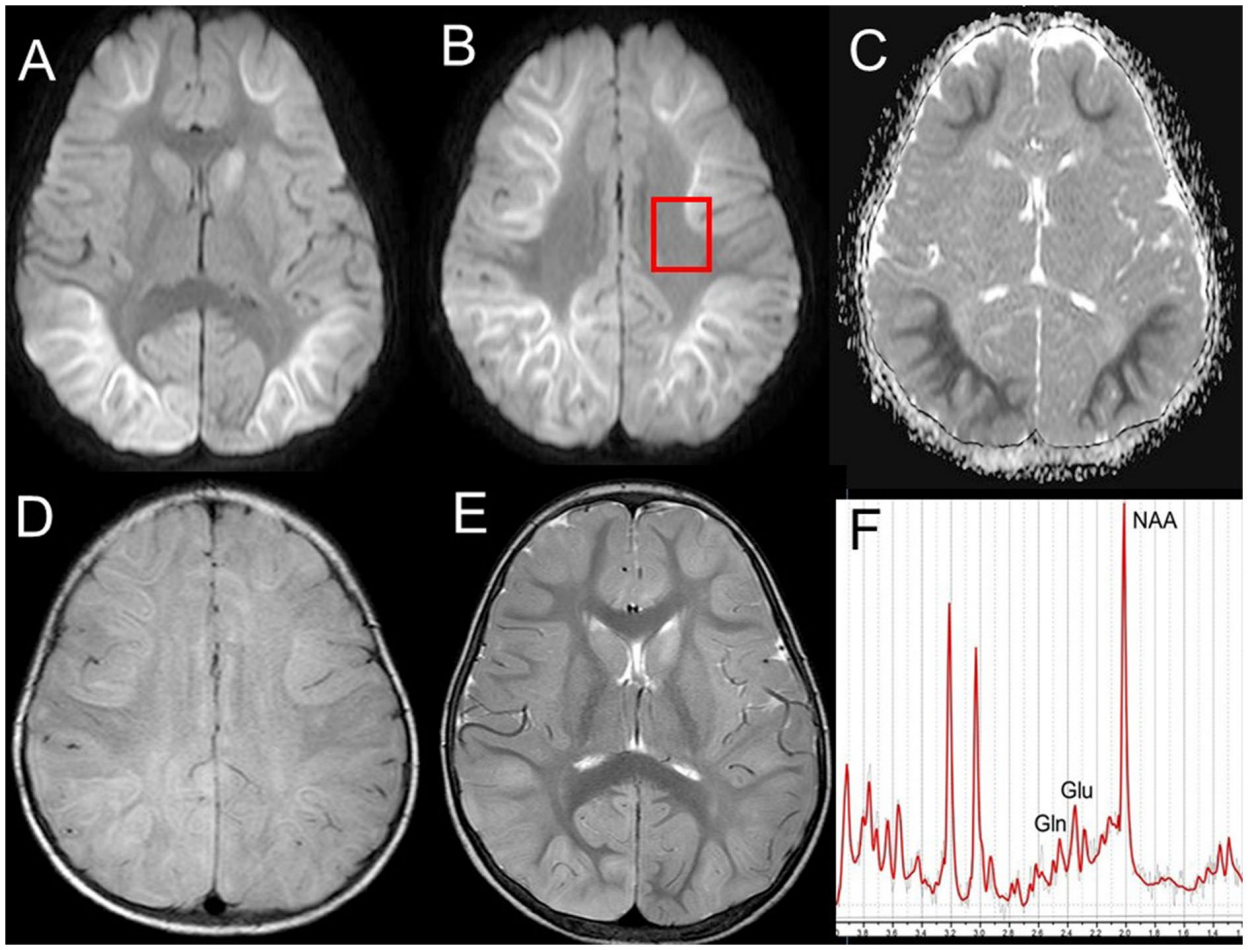
MRI and MRS on day 4 of the disease in a 1-year-old boy with AESD. DWI **(A,B)** showing high-signal lesions with reduced ADC **(C)** in the bilateral frontoparietal subcortical white matter (bright tree appearance) with central sparing. A box in **(B)** indicates the ROI of MRS. T2WI **(E)** showing cortical swelling and T2 prolongation. FLAIR imaging reveals additional U-fiber lesions **(D)**. MRS **(F)** [PRESS; TR/TE = 5000/30; ROI, the fronto-parietal white matter **(B)**] shows decreased NAA (5.45 mM/L; mean ± standard deviation, 6.8 ± 0.5) and increased glutamine (4.07 mM/L; 2.0 ± 0.3) (From Takanashi (2021) Acute encephalopathy with biphasic seizures and late reduced diffusion (in Japanese). Pediatrics of Japan 62, 939–947 with permission). MRI, magnetic resonance imaging; MRS, magnetic resonance spectroscopy; PRESS, point resolved spectroscopy; TR, repetition time; TE, echo time; ROI, region of interest; ADC, apparent diffusion coefficient; DWI, diffusion-weighted imaging; T2WI, T2-weighted imaging; AESD, acute encephalopathy with biphasic seizures and late reduced diffusion; FLAIR, fluid-attenuated inversion recovery; NAA, *N*-acetylaspartate.

MRI, especially DWI, is an imaging used to characterize AESD and is one of the diagnostic criteria for diagnosis (Mizuguchi et al., 2021). MRI performed on days 1–2 of the disease usually shows no abnormal lesions, including those on DWI. During days 3–9, DWI showed restricted diffusion in the subcortical white matter, the so-called bright tree appearance (BTA) (Takanashi et al., 2006a; Takanashi, 2009a; Takanashi, 2018; Mizuguchi et al., 2021) (Figures 2A–C). T2-weighted imaging (T2WI) and fluid-attenuated inversion recovery imaging also showed T2 prolongation in the subcortical white matter and a linear high intensity along the U-fibers (Figures 2D,E). Cortical hyperintensities on DWI, T2WI, and fluid-attenuated inversion recovery imaging were less prominent. The lesions were predominantly frontal or frontoparietal in location, sparing the peri-Rolandic region (central sparing). These are usually symmetric, but sometimes asymmetric or hemispheric; a diagnosis of hemiconvulsion hemiplegia epilepsy syndrome is often made in these patients. The BTA disappeared on follow-up MRI after 9 days; instead, high cortical intensity was often observed on DWI. Cerebral atrophy and T2 prolongation in the affected white matter are usually observed afterwards. FA and diffusion tensor imaging in AESD patients has not been reported previously.

#### 2.1.2. MERS

Reversible splenial lesions with transient reduced diffusion are observed in certain diseases and conditions, including high-altitude cerebral edema, rapid withdrawal of antiseizure medications, Kawasaki disease, X-linked Charcot–Marie–Tooth disease, myelin regulatory factor (*MYRF*) mutations, and encephalitis/encephalopathy (Starkey et al., 2017), which have been termed reversible splenial lesion syndrome (RESLES) and cytotoxic lesions of the corpus callosum (CLOCCs) (Blaauw and Meiners, 2020). Among them, a diagnosis of MERS is made for patients with encephalitis/encephalopathy whose MRI shows a reversible lesion of the corpus callosum involving at least the splenium (Tada et al., 2004; Takanashi, 2009a). MERS is the second most common subtype of encephalopathy syndrome (18%) in Japan, occurring in 80–140 children per year. Influenza virus (22%) is the most common pathogen, followed by rotavirus (9%), and HHV 6 and 7 (5%). The mean age for MERS is 5.6 years old, which is higher than those for ANE (2.5 years old) and AESD (1.6 years old) (Kasai et al., 2020). MERS typically presents with central nervous system symptoms such as delirious behavior, consciousness disturbances, and seizures, with complete recovery within a month. Serum hyponatremia is also often observed (Takanashi et al., 2009b). Bacterial infections account for 2% of MERS cases, most of which are urinary tract infections, particularly acute focal bacterial nephritis (Okada et al., 2022). When an older child with a strong inflammatory response exhibits delirium or consciousness disturbance, it is necessary to perform an MRI to check for this condition.

**Figure 3:**
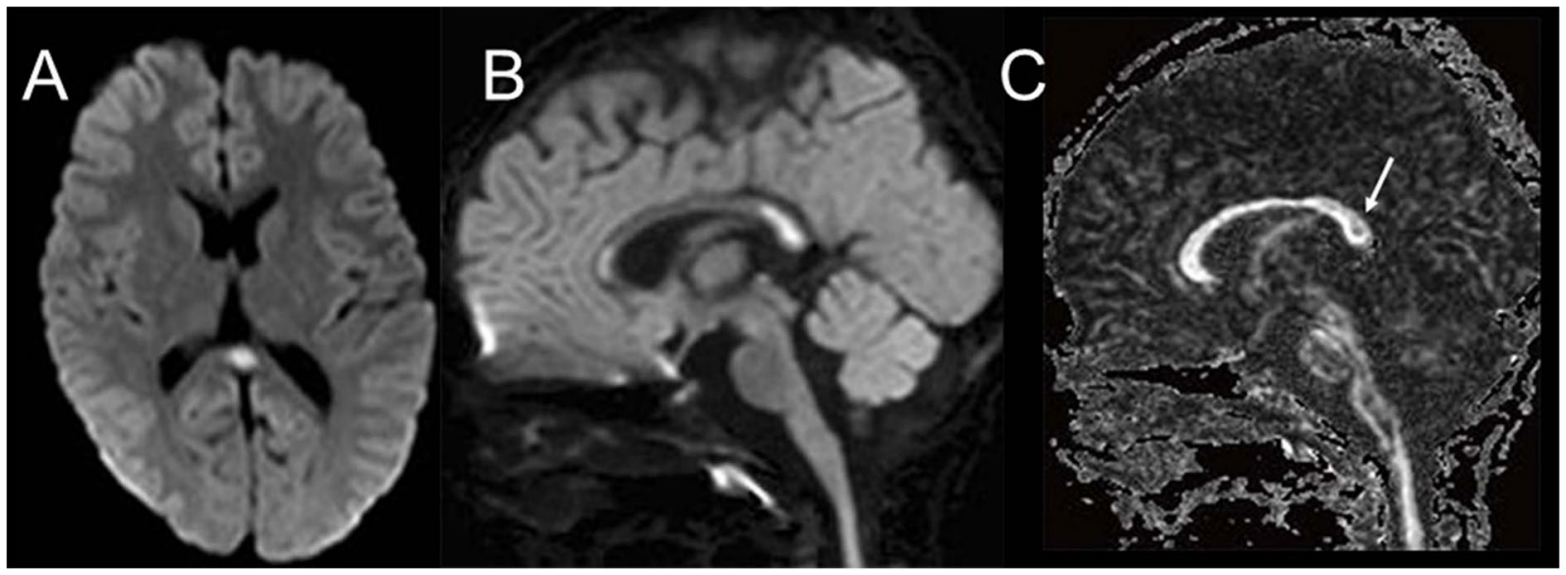
MRI in a teenager with MERS type 1. DWI **(A,B)** show a high-signal lesion in the splenium and genu of the corpus callosum, with reduced diffusion. The FA map shows reduced FA **(C)**, all of which disappeared on the follow-up MRI. MERS, clinically mild encephalitis/encephalopathy with reversible splenial lesions; DWI, diffusion-weighted imaging; FA, fractional anisotropy; MRI, magnetic resonance imaging.

**Figure 4:**
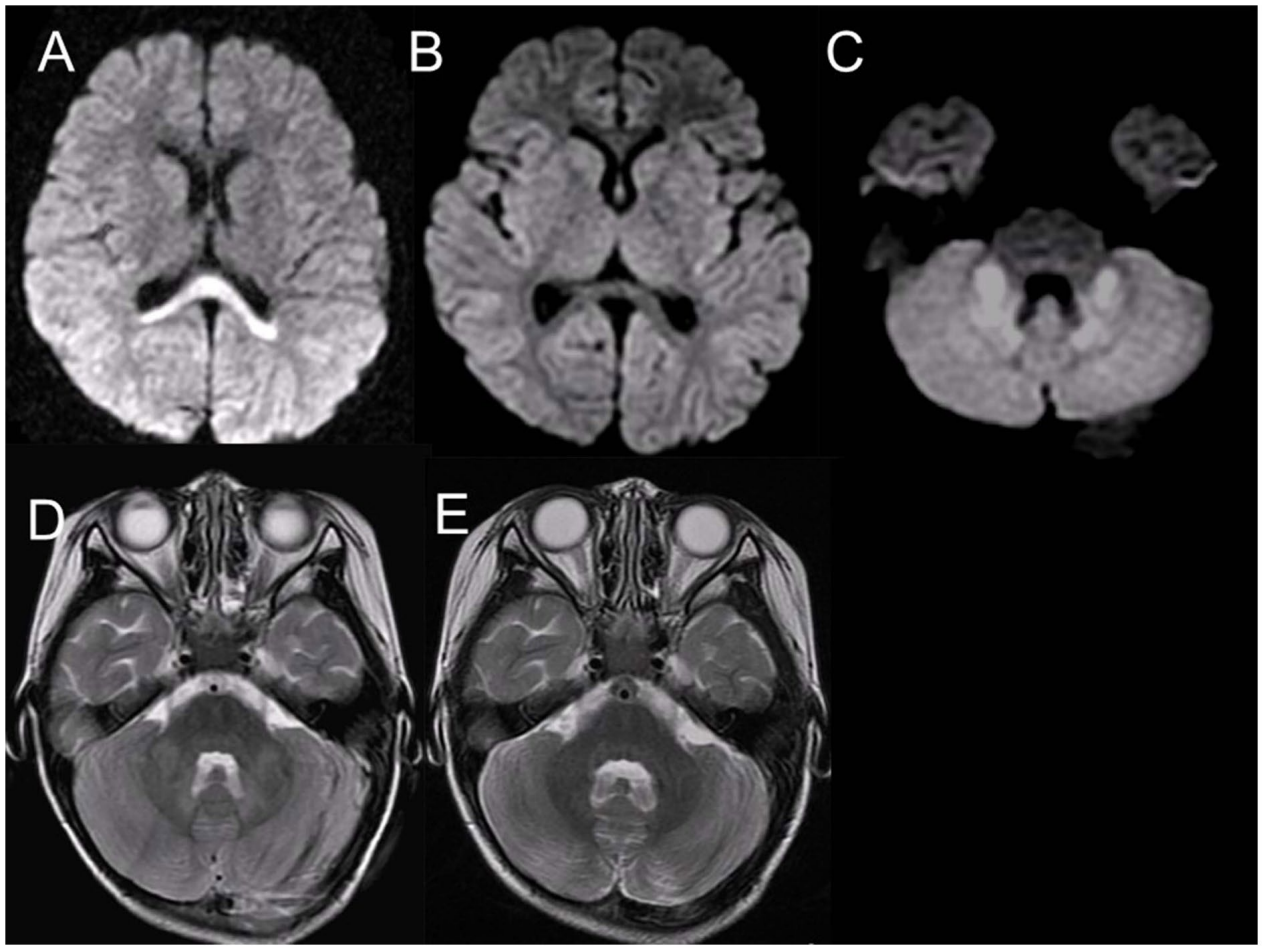
MRI of 3-year-old girl with cerebellitis associated with rotavirus. DWI on day 4 **(A)** shows a splenial lesion compatible with type 1 MERS, which disappeared on day 6 **(B)**. DWI on day 6 shows abnormally reduced diffusion in the bilateral middle cerebellar peduncles and nuclei **(C)**. T2WI also shows mild hyperintensity in the cerebellar cortex in addition to the middle cerebellar peduncle and nuclear lesions **(D)**. MRI on day 65 shows an almost normal cerebellum, other than mild atrophy **(E)** (from Takanashi et al., 2010b with permission). MRI, magnetic resonance imaging; DWI, diffusion-weighted imaging; MERS, clinically mild encephalitis/encephalopathy with reversible splenial lesions; T2WI, T2-weighted imaging.

DWI is a key imaging modality for the diagnosis of MERS and shows homogeneously reduced diffusion in the corpus callosum with no contrast enhancement (Table 1). Type 1 MERS involves the corpus callosum, at least the splenium (Figures 3A,B), whereas type 2 MERS has symmetrical white matter lesions in addition to the corpus callosum (Takanashi et al., 2006b; Takanashi, 2009a; Takanashi, 2018). The time course differed between the splenial and white matter lesions, wherein the latter disappeared earlier than the former (Takanashi et al., 2010a). Previous studies have reported a decrease in FA or normal FA for both structures (Shimizu et al., 2007; Osuka et al., 2010; Zhu et al., 2016; Fong et al., 2017). Our patient with type 1 MERS showed decreased ADC and FA values in the splenium (Figure 3C). Some patients with MERS associated with rotavirus gastroenteritis subsequently develop cerebellitis, who clinically present with disorders of consciousness as the initial neurological symptom, followed by mutism, and have residual mild cerebellar symptoms. MRI shows a reversible splenial lesion in the acute stage, abnormal signal intensity in the cerebellar white matter/nuclei in the acute-to-subacute stages, followed by increased signal in the cerebellar cortex, and finally cerebellar atrophy (Takanashi et al., 2010b) (Figure 4).

Familial and/or recurrent type 2 MERS suggest the presence of genetic factors. Whole-exome sequencing has revealed mutations of the *MYRF* gene in these patients (Kurahashi et al., 2018). *MYRF* is a transcriptional regulator required for oligodendrocyte differentiation and myelin maintenance. Functional defects in *MYRF* are likely to be causally associated with encephalopathy with extensive myelin vacuolization. These findings strongly suggest that the transient reduced diffusion observed on DWI results from the intramyelinic edema.

#### 2.1.3. ANE

ANE is a fulminant type of encephalopathy in which a cytokine storm has been postulated as the pathological mechanism. ANE presents with convulsions, coma, and signs of multi-organ involvement. The influenza virus (34%) was the most common pathogen, followed by HHV 6 and 7 (16%). The outcomes of patients with ANE remain poor, with a mortality rate of up to 25% (Kasai et al., 2020).

**Figure 5:**
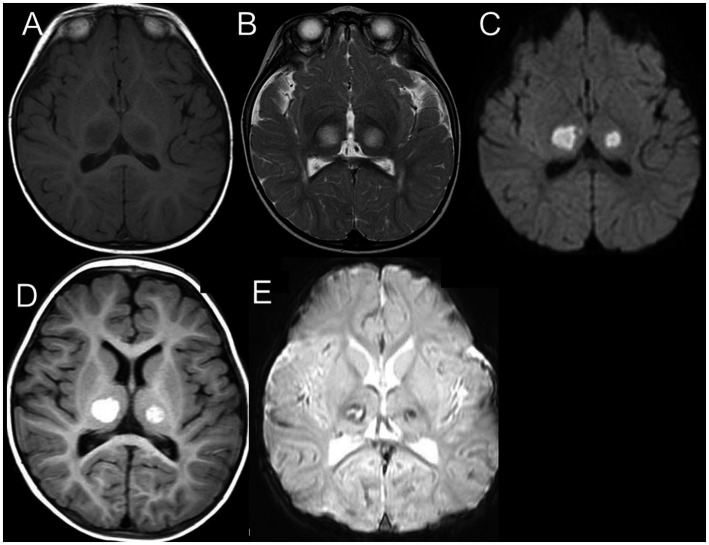
MRI of 1-year-old boy with ANE. MRI on day 4 reveals bilateral symmetric thalamic lesions [low on T1WI **(A)** and high on T2WI **(B)**] with reduced diffusion **(C)**. MRI on day 14 shows high intensity on T1WI **(D)** with partly low intensity on T2 star-weighted imaging **(E)**, suggesting hemorrhagic degeneration (from Takanashi, 2018 with permission). MRI, magnetic resonance imaging; ANE, acute necrotizing encephalopathy; T1WI, T1-weighted imaging; T2WI, T2-weighted imaging.

In the acute stage, computed tomography or MRI showed diffuse cerebral edema and symmetric and multifocal lesions (low density on CT and T1 low/T2 high on MRI) in the thalamus (Figures 5A,B) and other central nervous system regions, including the posterior limb of the internal capsule, posterior putamen, cerebral and cerebellar deep white matter, and the upper brainstem tegmentum (Mizuguchi et al., 2007, 2021; Takanashi, 2018) (Table 1). DWI revealed reduced diffusion of the acute thalamic lesions (Figure 5C), which is useful for the differential diagnosis of acute disseminated encephalomyelitis, usually with increased diffusion. Thalamic lesions often show hemorrhagic degeneration and cystic changes. Reflecting petechial hemorrhage, T1-weighted imaging (T1WI) showed high-signal ring-like or circular high-signal lesions in the thalamus after 3 days, wherein some showed a low signal on T2WI (Figures 5D,E).

### 2.2. ASL

ASL can noninvasively assess cerebral perfusion using labeled blood as an endogenous tracer. ASL does not require radiation exposure or the use of contrast agents and can be repeated, making it suitable for evaluating cerebral perfusion in pediatric emergencies (Kitajima and Uetani, 2023). ASL has been reported to be useful in evaluating cerebral perfusion in acute encephalitis (Noguchi et al., 2016), epilepsy (Matsuura et al., 2015; Shimogawa et al., 2017), and migraines (Uetani et al., 2018).

A consensus statement from the ISMRM Perfusion Study Group and the European ASL in the Dementia Consortium on the practical application of ASL in clinical practice recommends the use of a 3-T unit, pseudo-continuous ASL labeling, background suppression, segmented 3D readout, no use of vascular crushing gradients, calculation and presentation of both label and control difference images, and cerebral blood flow in absolute units using a simplified model (Alsop et al., 2015). In terms of the labeling method, pseudo-continuous ASL uses a narrow labeling plane and a train of very short radio-frequency pulses, providing high efficiency and inducing a higher signal-to-noise ratio and reproducibility than pulsed ASL (Dai et al., 2008). The postlabeling delay/inversion time is the time between labeling and signal acquisition. This is a critical factor for obtaining accurate perfusion information in ASL. The recommendations for postlabeling delay/inversion time are 2,000 ms for neonates and 1,500 ms for children (Alsop et al., 2015).

#### 2.2.1. AESD

**Figure 6:**
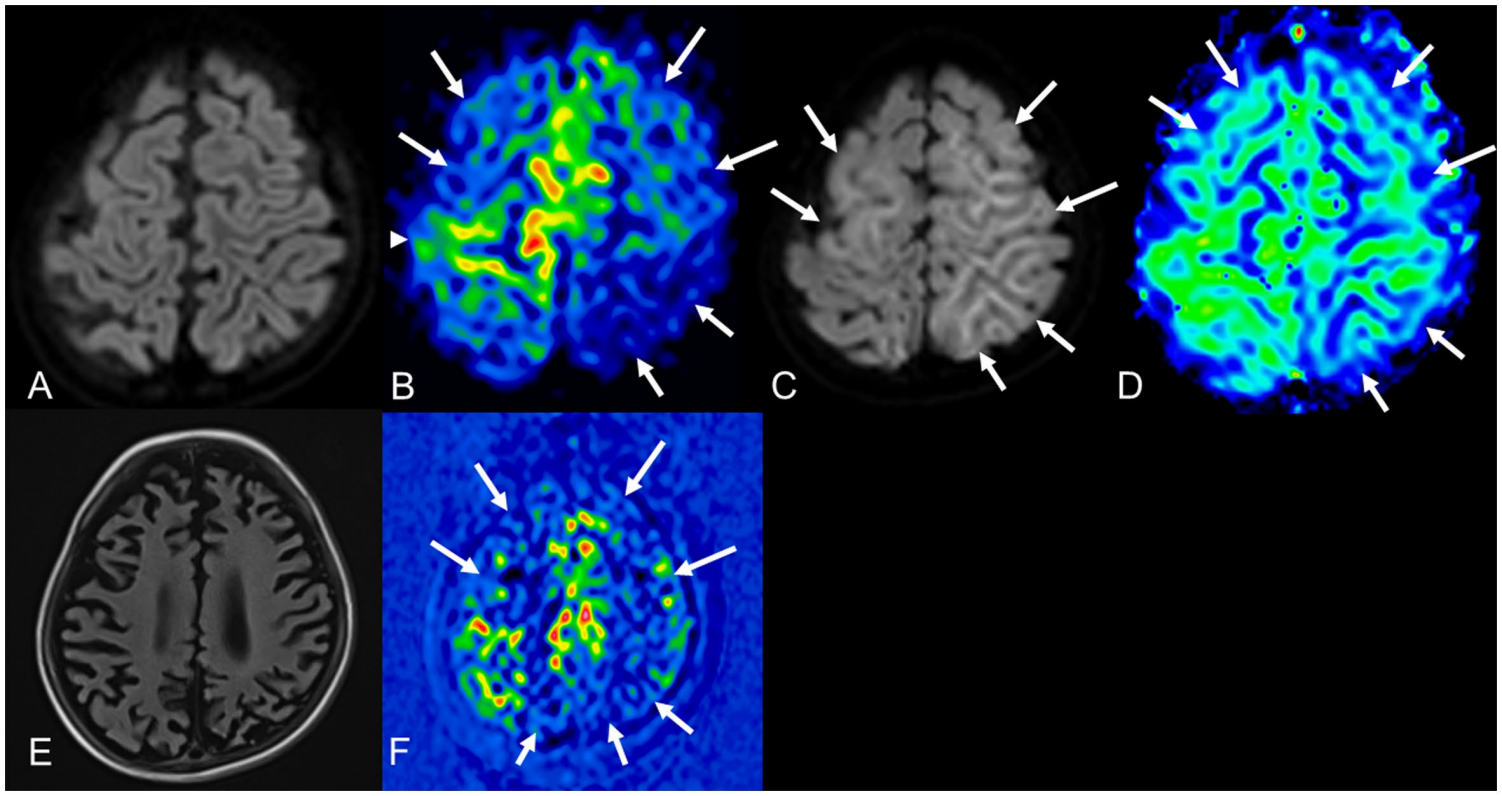
A 34-month-old AESD female with residual psychomotor retardation and epilepsy. DWI 8.5 h after early seizure **(A)** shows normal, but pulsed ASL **(B)** shows severe hypoperfusion (arrows) in the bilateral frontal and left parietal cortex with central sparing (arrowhead). She underwent targeted temperature management under continuous sedation and ventilation, and late seizures did not occur. DWI 5 days after early seizure **(C)** shows hyperintensity in the bilateral frontal and left parietal cerebral subcortical white matter (BTA, arrows). The distribution of BTA is consistent with the area exhibiting hypoperfusion on ASL after early seizure. Pulsed ASL 5 days after early seizure **(D)** shows slight hypoperfusion (BTA, arrows). FLAIR imaging **(E)** and pulsed ASL **(F)** 6 months later showed severe atrophy and hypoperfusion in bilateral frontal and parietal lobes (arrows). AESD, acute encephalopathy with biphasic seizures and late reduced diffusion; DWI, diffusion-weighted imaging; ASL, arterial spin labeling; BTA, bright tree appearance; FLAIR, fluid-attenuated inversion recovery.

**Figure 7:**
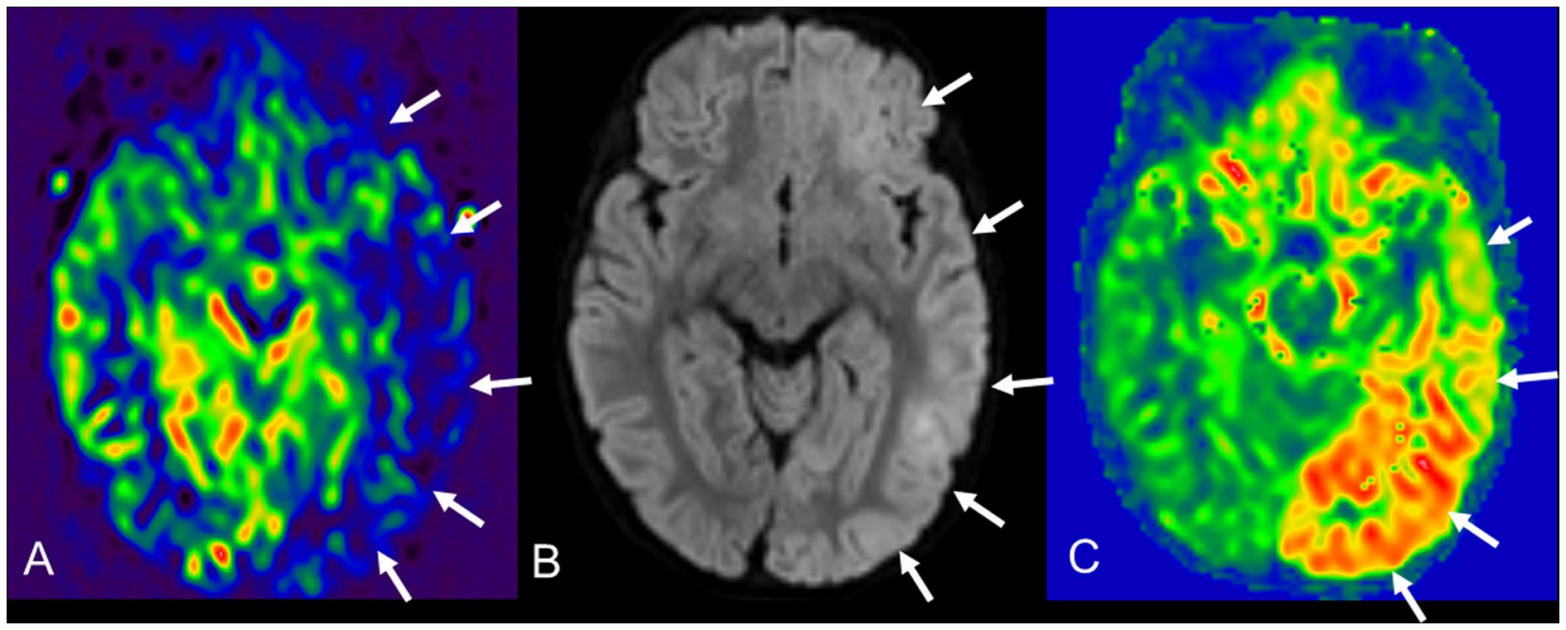
A 22-month-old AESD male with residual psychomotor retardation. On day 1, DWI and other conventional sequences show normal findings, but pulsed ASL **(A)** shows moderate hypoperfusion in the left temporal, occipital, and frontal cortex asymmetry (arrows). He was treated with targeted temperature management with ventilator and sustained sedation. Late seizures did not occur owing to treatment, but DWI **(B)** and pulsed ASL **(C)** 5 days after early seizures show apparent BTA and moderate hyperperfusion (arrows) in the left subcortical region, where ASL exhibited hypoperfusion on day 1. AESD, acute encephalopathy with biphasic seizures and late reduced diffusion; DWI, diffusion-weighted imaging; ASL, arterial spin labeling; BTA, bright tree appearance.

Changes in cerebral perfusion during AESD have been reported on single-photon emission computerized tomography scan or ASL in several cases (Yamanouchi and Mizuguchi, 2006; Kuya et al., 2017; Yokoyama et al., 2017; Uetani et al., 2020). Chronological observations of cerebral perfusion in patients with AESD using ASL showed hypoperfusion between 8.5 and 22 h after early seizures, and hyperperfusion within 24 h after late seizures (Uetani et al., 2020) (Figures 6B, 7A,C). Notably, the distribution of perfusion abnormalities on ASL was consistent with that of BTA on DWI with central sparing (Uetani et al., 2020) (Figures 6B,C, 7A,B). ASL after early seizures can be useful in predicting the onset of AESD because conventional MR sequences, including DWI (Figure 6A), do not show abnormalities on days 1–2. Some patients have only faint BTA on DWI on days 3–9, and others may not have BTA because MRI cannot be performed at the appropriate time due to lack of late seizures caused by sedation with targeted temperature management. Even in these cases, the evaluation combined with hyperperfusion on ASL may enhance a diagnostic potential of AESD (Figures 7B,C). During the subacute phase, more than 3 days after late seizures, various perfusion patterns were observed, including mild hyperperfusion to hypoperfusion, compared to normal brain perfusion regions (Uetani et al., 2020) (Figure 6D). In the chronic phase, 6 months after late seizures, 70% (9/13) of patients showed hypoperfusion on ASL (Uetani et al., 2020) (Figure 6F). The pathological mechanism of hypoperfusion after early seizures has not been elucidated; however, previous studies in drug-resistant focal epilepsy have suggested that postictal hypoperfusion occurs approximately 1–1.5 h after seizures, and vasoconstriction due to glutamate, cyclooxygenase-2, and L-type calcium channels may contribute to perfusion abnormalities (Farrell et al., 2017; Gaxiola-Valdez et al., 2017). Although no AESD patients have been reported to have hypoperfusion on ASL earlier than 8.5 h after early seizures, further studies are needed to determine whether hypoperfusion can be detected within a few hours of early seizures, as this would be more useful in the very early diagnosis of AESD. Differentiating AESD from febrile seizures is difficult in early seizures but is very important. This is because the neurological outcome after a prolonged febrile seizure is usually good. However, AESD often leaves patients with mental deficits and/or epilepsy. We encountered a pediatric case with a cluster of febrile seizures with no abnormal perfusion on ASL on day 2 and no development of AESD in the subsequent course. Hypoperfusion with central sparing approximately 20 h after early seizure may be useful in differentiating AESD from febrile seizures, but future prospective studies are required.

#### 2.2.2. MERS, ANE, and cerebellitis

To the best of our knowledge, there have been no reports regarding perfusion abnormalities in ASL for MERS or ANE. In our experience, several patients with MERS do not show abnormal perfusion in ASL, including the corpus callosum region.

**Figure 8:**
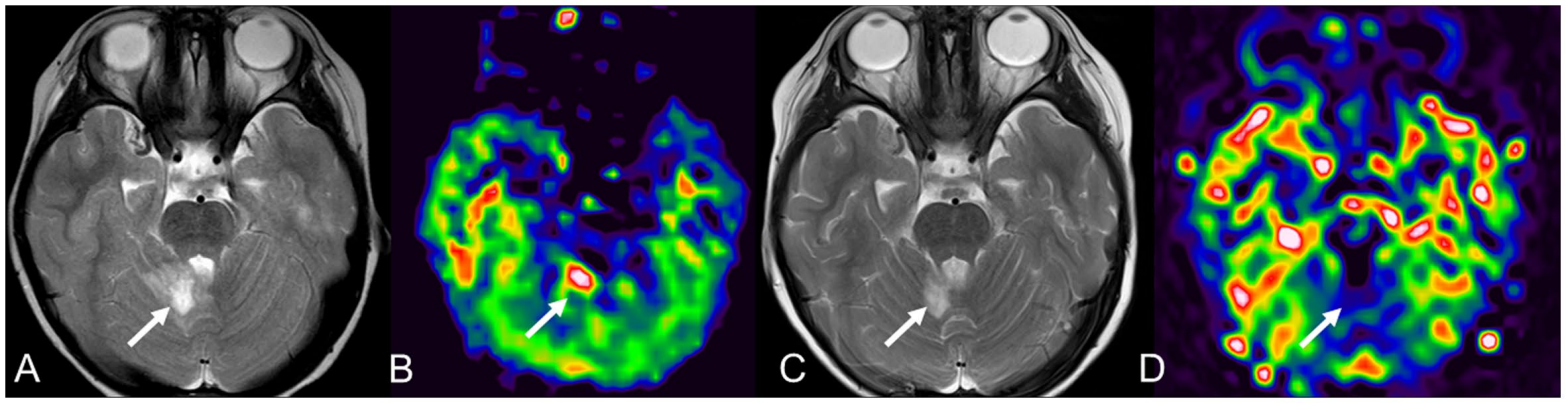
A 3-year-old female with cerebellitis associated with myelin oligodendrocyte glycoprotein-immunoglobulin G-associated disease without neurological sequelae. She has hyperintensity on T2WI **(A, arrow)** and hyperperfusion on ASL **(B, arrow)** in the right cerebellum on day 7. Follow-up MRI on day 18 shows a slight decrease in the area of hyperintensity on T2WI **(C, arrow)** and hypoperfusion on ASL **(D, arrow)**. T2WI, T2-weighted imaging; ASL, arterial spin labeling; MRI, magnetic resonance imaging.

A previous ASL study on central nervous system infections reported hyperperfusion in two pediatric patients with rotavirus or staphylococcal infections (Noguchi et al., 2016). At our institution, two pediatric patients with cerebellitis associated with myelin oligodendrocyte glycoprotein immunoglobulin G-associated disease or influenza B virus infection exhibited hyperperfusion on ASL in the cerebellar lesion in the acute phase (Figure 8). A 4-year-old boy with influenza B virus infection had restricted diffusion and hyperperfusion in the bilateral cerebellar hemispheres and cytotoxic lesions of the corpus callosum on DWI with normal perfusion on ASL on day 2 of encephalopathy.

### 2.3. MRS

Proton MRS reveals the chemical shifts of a large number of biologically relevant metabolites, and allows noninvasive exploration of tissue metabolism *in vivo*, providing neurophysiological and neurochemical information. Short echo time (TE) spectra (TE <35 ms) allow the assessment of many metabolites, even those with short T2 values, and are useful for evaluating children with metabolic or uncertain pathological mechanism. MR visible metabolites include *N*-acetylaspartate (NAA) at 2.02 ppm as neuro-axonal marker, choline (Cho) at 3.19 ppm as marker for oligodendrocyte or myelin membrane, creatine (Cr) at 3.04 ppm as energy marker, myo-Inositol (mIns) at 3.55 ppm as astrocytic marker, glutamate (Glu) at 2.35 ppm as excitotoxic or neuronal marker, glutamine (Gln) at 2.45 ppm as astrocytic marker or osmolyte, and lactate at 1.33 ppm as marker for anaerobic glycolysis or mitochondrial dysfunction. LCModel has recently been used for the quantification of metabolites instead of semi-quantitative analysis using relatively stable Cr as the denominator.

#### 2.3.1. AESD

The exact pathogenesis of AESD is uncertain; however, excitotoxic injury with delayed or apoptotic neuronal death is hypothesized to be a possible mechanism, based on MRS (Takanashi et al., 2009c, 2015). MRS (point resolved spectroscopy; repetition time/TE = 5,000/30; region of interest, fronto-parietal white matter shown in Figure 2B; volume of interest, 15 × 15 × 20mm^3^) revealed an increase in Glu levels on days 1–4, followed by an increase in Gln on days 3–12 (Figure 2F). Glutamatergic neurons release Glu into the synaptic cleft, where it is taken up by the surrounding astrocytes. Glu taken up by nearby astrocytes is amidated to a harmless compound, Gln, by glutamine synthetase, which is located only in astrocytes, and returned to the neurons for reuse as Glu, completing the Glu-Gln cycle. Acute Glu elevation followed by subacute Gln elevation observed in MRS may reflect the process by which excess Glu is released beyond the astrocyte processing capacity. From a clinical perspective, Glu or Gln elevation before BTA is useful for the early differential diagnosis of AESD and prolonged febrile seizures, and may contribute to early therapeutic intervention. MRS also showed a decrease in NAA, a marker of neuroaxonal function, within 1 week of onset in AESD with sequelae. On the other hand, it is nearly normal in those without sequelae, suggesting that MRS may be predictive of outcome in AESD (Takanashi et al., 2020).

#### 2.3.2. MERS

**Figure 9:**
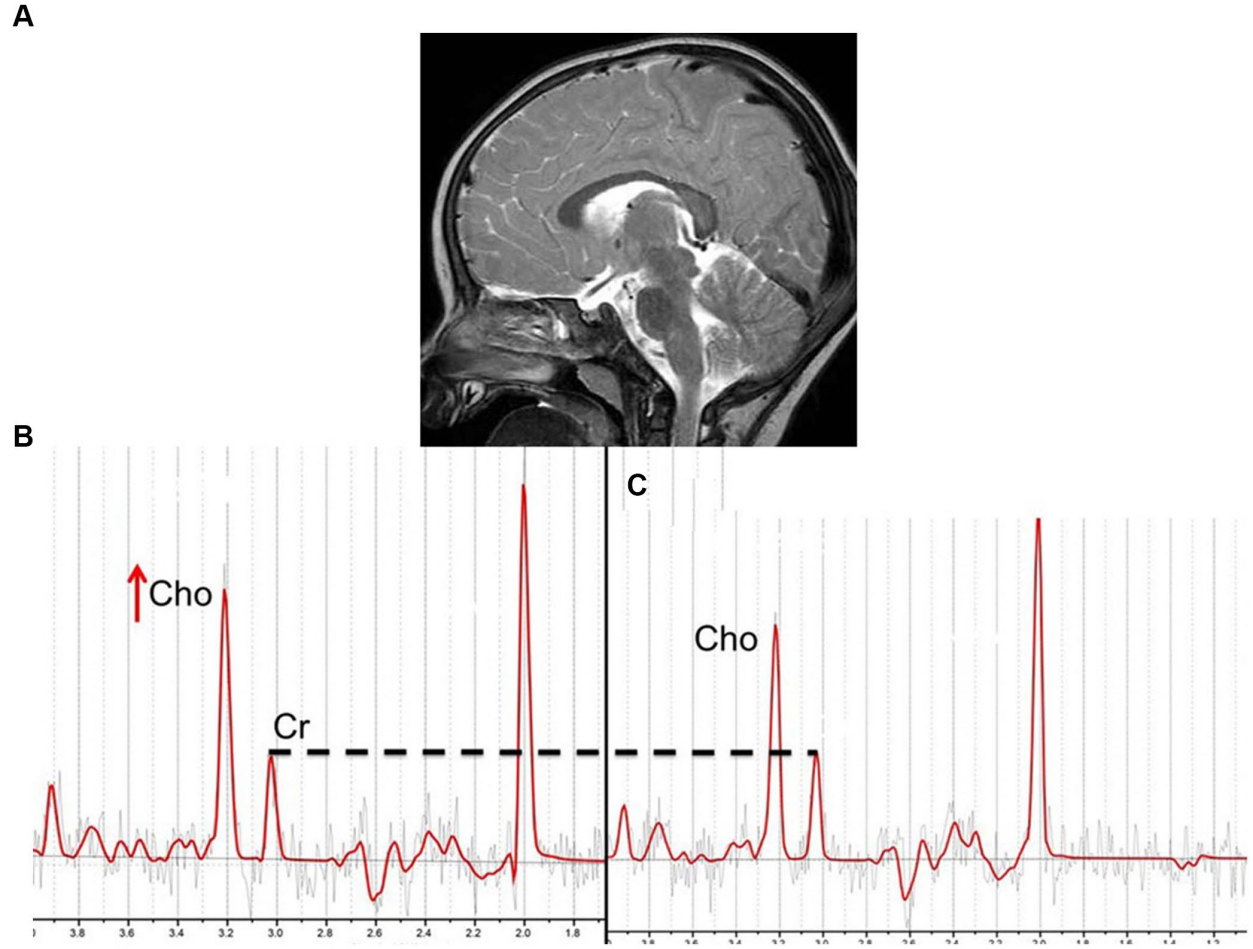
Sagittal T2WI on day 4 **(A)**, and MRS (PRESS; TR/TE = 3000/144; ROI, the splenium of the corpus callosum) on days 4 and 28 **(B,C)** in a 1-year-old female with MERS type 1. Sagittal T2WI on day 4 revealed a high-signal intensity lesion in the splenium of the corpus callosum (MERS type 1). MRS on day 4 showed an increased Cho/Cr ratio (0.90), which normalized by day 28 (Cho/Cr = 0.73), when the splenial lesion disappeared. T2WI, T2-weighted imaging; MRS, magnetic resonance spectroscopy; PRESS, point resolved spectroscopy; TR, repetition time; TE, echo time; ROI, region of interest; MERS, clinically mild encephalitis/encephalopathy with reversible splenial lesions; Cho, choline; Cr, creatinine.

MRS for MERS has only been reported in a few patients, and the results have been variable. The Cho/Cr ratio in the splenium of a patient with type 1 MERS was reported to be normal on multivoxel MRS (Shimizu et al., 2007). Another patient with restricted diffusion in the corpus callosum and white matter showed normal NAA/Cr and Cho/Cr on single-voxel MRS with a short TE; however, T2 prolongation in the lesions persisted over time (Ueda et al., 2014), which is atypical for MERS. In contrast, two patients with type 1 MERS showed an increased Cho/Cr ratio in the splenium on single-voxel MRS with short and long TE, which normalized on follow-up MRS (Anbe et al., 2017) (Figure 9). The reversible increase in Cho levels in the splenium may reflect intramyelinic edema, which is hypothesized to occur in patients with *MYRF* mutations. Nevertheless, it is necessary to perform MRS in more patients to elucidate the pathophysiology of MERS.

#### 2.3.3. ANE

Only a few MRS findings in the thalamic lesion of ANE have been reported, which show an increased lactate-lipid peak at approximately 1.3 ppm, and an increased Glu/Gln complex with normal or decreased NAA. MRS findings may reflect necrosis, anaerobiosis, or excitotoxicity (Goo et al., 2003; Aydin et al., 2010; Wertheimer et al., 2022). The signal-to-noise ratio of MRS in thalamic lesions is sometimes poor due to petechial hemorrhage.

## 3. Conclusion

Information on MRI in pediatric acute encephalopathy syndromes has been described. Elucidating the pathogenesis and establishing treatments, especially for AESD, are major issues for the future, wherein advances in neuroradiology will help solve them.

## Author contributions

J-iT and HU conceptualized and designed the manuscript, researched the data for review, wrote and revised the manuscript, contributed to the discussions. All authors contributed to the article and approved the submitted version.

## Funding

This study was funded by a Grant-in-Aid for Research on Measures for Intractable Diseases No. 21FC1005 (J-iT) from the Japanese Ministry of Health, Labour and Welfare; JSPS KAKENHI Grant Numbers JP19K08237 and JP23K07192 (J-iT).

## Conflict of interest

The authors declare that the research was conducted in the absence of any commercial or financial relationships that could be construed as a potential conflict of interest.

## Publisher’s note

All claims expressed in this article are solely those of the authors and do not necessarily represent those of their affiliated organizations, or those of the publisher, the editors and the reviewers. Any product that may be evaluated in this article, or claim that may be made by its manufacturer, is not guaranteed or endorsed by the publisher.

## Glossary

ADC: Apparent diffusion coefficient
AESD: Acute encephalopathy with biphasic seizures and late reduced diffusion
ANE: Acute necrotizing encephalopathy
ASL: Arterial spin labeling
BTA: Bright tree appearance
Cho: Choline
Cr: Creatine
DWI: Diffusion-weighted imaging
FA: Fractional anisotropy
FLAIR: Fluid-attenuated inversion recovery
Gln: Glutamine
Glu: Glutamate
HHV: Human herpes virus
MERS: Clinically mild encephalitis/encephalopathy with reversible splenial lesions
mIns: myo-Inositol
MR: Magnetic resonance
MRI: Magnetic resonance imaging
MRS: Magnetic resonance spectroscopy
MYRF: Myelin regulatory factor
NAA: *N*-acetylaspartate
T1WI: T1-weighted imaging
T2WI: T2-weighted imaging
TE: Echo time
